# GMP-Compliant Production of Autologous Adipose-Derived Stromal Cells in the NANT 001 Closed Automated Bioreactor

**DOI:** 10.3389/fbioe.2022.834267

**Published:** 2022-03-09

**Authors:** Joan C. Fitzgerald, Niamh Duffy, Giacomo Cattaruzzi, Francesco Vitrani, Alice Paulitti, Flavia Mazzarol, Prisca Mauro, Antonio Sfiligoj, Francesco Curcio, Deirdre M. Jones, Veronica McInerney, Janusz Krawczyk, Jack Kelly, Andrew Finnerty, Katya McDonagh, Una McCabe, Matthew Duggan, Lauren Connolly, Georgina Shaw, Mary Murphy, Frank Barry

**Affiliations:** ^1^ Regenerative Medicine Institute (REMEDI), National University of Ireland Galway, Galway, Ireland; ^2^ VivaBioCell S.p.A., Udine, Italy; ^3^ Dipartimento di Area Medica (DAME), University of Udine, Udine, Italy; ^4^ Department of Plastic and Reconstructive Surgery, Roscommon University Hospital, Galway, Ireland; ^5^ HRB Clinical Research Facility, National University of Ireland Galway, Galway, Ireland; ^6^ Department of Haematology, Galway University Hospital, Galway, Ireland; ^7^ Department of Plastic and Reconstructive Surgery, Galway University Hospital, Galway, Ireland; ^8^ Centre for Cell Manufacturing Ireland, National University of Ireland Galway, Galway, Ireland

**Keywords:** mesenchymal stromal cells, automation, GMP—good manufacturing practice, autologous, bioreactor

## Abstract

In recent years mesenchymal stromal cells (MSCs) have received a great deal of interest for the treatment of major diseases, but clinical translation and market authorization have been slow. This has been due in part to a lack of standardization in cell manufacturing protocols, as well as a lack of biologically meaningful cell characterization tools and release assays. Cell production strategies to date have involved complex manual processing in an open environment which is costly, inefficient and poses risks of contamination. The NANT 001 bioreactor has been developed for the automated production of small to medium cell batches for autologous use. This is a closed, benchtop system which automatically performs several processes including cell seeding, media change, real-time monitoring of temperature, pH, cell confluence and cell detachment. Here we describe a validation of the bioreactor in an environment compliant with current good manufacturing practice (cGMP) to confirm its utility in replacing standardized manual processing. Stromal vascular fraction (SVF) was isolated from lipoaspirate material obtained from healthy donors. SVF cells were seeded in the bioreactor. Cell processing was performed automatically and cell harvesting was triggered by computerized analysis of images captured by a travelling microscope positioned beneath the cell culture flask. For comparison, the same protocol was performed in parallel using manual methods. Critical quality attributes (CQA) assessed for cells from each process included cell yield, viability, surface immunophenotype, differentiation propensity, microbial sterility and endotoxin contamination. Cell yields from the bioreactor cultures were comparable in the manual and automated cultures and viability was >90% for both. Expression of surface markers were consistent with standards for adipose-derived stromal cell (ASC) phenotype. ASCs expanded in both automated and manual processes were capable of adipogenic and osteogenic differentiation. Supernatants from all cultures tested negative for microbial and endotoxin contamination. Analysis of labor commitment indicated considerable economic advantage in the automated system in terms of operator, quality control, product release and management personnel. These data demonstrate that the NANT 001 bioreactor represents an effective option for small to medium scale, automated, closed expansion of ASCs from SVF and produces cell products with CQA equivalent to manual processes.

## Introduction

Mesenchymal stromal cells (MSCs) have attracted a great deal of interest in recent years because of their therapeutic potential in addressing a number of major diseases. The MSC as a cellular entity was first described almost 5 decades ago by Friedenstein and others, who isolated the cells from rat bone marrow and showed that they were capable of supporting haematopoiesis. The MSC as a therapeutic entity was proposed by Caplan et al. some 3 decades ago (Wakitani et al., 1994), and the first MSC investigative treatment of patients was reported in 1995 (Lazarus et al., 1995).

Despite the long history of investigation and large numbers of clinical trials either underway or completed, the number of market authorizations is still low. Although many early phase clinical trials are listed in registries, only a tiny fraction of these proceed to later stage testing and then fewer still receive approval. There are many reasons for this lack of progression of a successful pipeline and foremost amongst these is the challenge associated with standardized and consistent manufacturing as well as the lack of biologically meaningful cell characterization tools and standard release assays. It goes without saying that highly controlled manufacturing processes are essential to ensure that variability between batches is minimal. This has been one of the extraordinary flaws in the MSC field—cell expansion protocols have generally relied on manual processing in an open environment with a great deal of intrinsic variability including cell source, initial isolation, expansion media, use of supplements etc. (Kirouac and Zandstra, 2008). Although MSCs are widely used as an allogeneic product, there is strong interest in autologous application and many trials of autologous MSCs are listed in NIH clinical trials registry. These include applications in cardiac failure (Noiseux et al., 2016; Hare et al., 2017; Barry, 2019), renal transplantation (US National Library of Medicine, 2021a), ALS (US National Library of Medicine, 2021b) as well as a number of trials currently underway to test the effectiveness of autologous MSC treatment in acute respiratory distress associated with SARS-CoV-2.

In the production of MSCs for autologous use, the impact of processing variables is more acute than in allogeneic use. It is imperative that standardized, highly controlled and automated processing solutions are employed. Here we provide one solution to address the manufacturing bottleneck, namely the use of the highly automated NANT 001 bioreactor, developed for automated production of small to medium scale cell batches. It is a benchtop-sized, closed system which automatically performs several processes including cell seeding, media changes, real-time monitoring of temperature, pH, imaging and estimating cell confluency. Our interest was in providing a validation of the NANT 001 bioreactor in a fully GMP environment to confirm its utility in replacing standardized manual processing. For this purpose, a manual GMP-compliant process was converted to an automated protocol for the expansion of ASCs using the NANT 001 bioreactor. Our intention was to provide a comparison between the two processes to justify adoption of the bioreactor manufacturing system in clinical production.

The result of this study indicated that 1) the NANT 001 bioreactor was fully adaptable for use in regulated GMP-compliant manufacturing, 2) the cell product was comparable to that obtained using conventional methods, 3) the bioreactor provided significant advantages in terms of labor commitment and 4) it significantly reduced the cost of manufacture. Based on these observations we conclude that automated cell expansion using the bioreactor represents a highly significant advantage over conventional manual methods.

## Materials and Methods

### Bioreactor Design

The NANT 001 bioreactor system uses a sterile, single-use cartridge consisting of a 636 cm^2^ cell-culture chamber connected in a closed system to a series of bags containing complete culture medium (CCM), wash buffer, detaching agent, cell suspension for seeding, a waste bag and a harvesting bottle. Pinch-tube valves and a peristaltic pump control speed and volume of liquid import and export. The tilt and shake mechanisms of the cell culture chamber ensure homogenous distribution of reagents inside the culture chamber, allow complete emptying of culture medium and promote cell detachment. Continuous monitoring of temperature, pH and cell confluency is performed throughout culture. A web application allows remote monitoring of these critical parameters. For confluency estimation, an integrated, self-operating and auto-focusing microscope acquires images at given intervals. Images are automatically processed by an integrated software for the cell confluency estimation. This can be used to prompt media replenishment at a user-specified confluency or to identify the optimal time for cell harvesting. Automated cell detachment, harvesting and exporting to a collection bottle for preparation of the final formulation is also performed. At the end of the process, a detailed cell culture report file is generated which details all critical parameters and operator interactions ensuring complete traceability of the process and any deviations.

**FIGURE 1:**
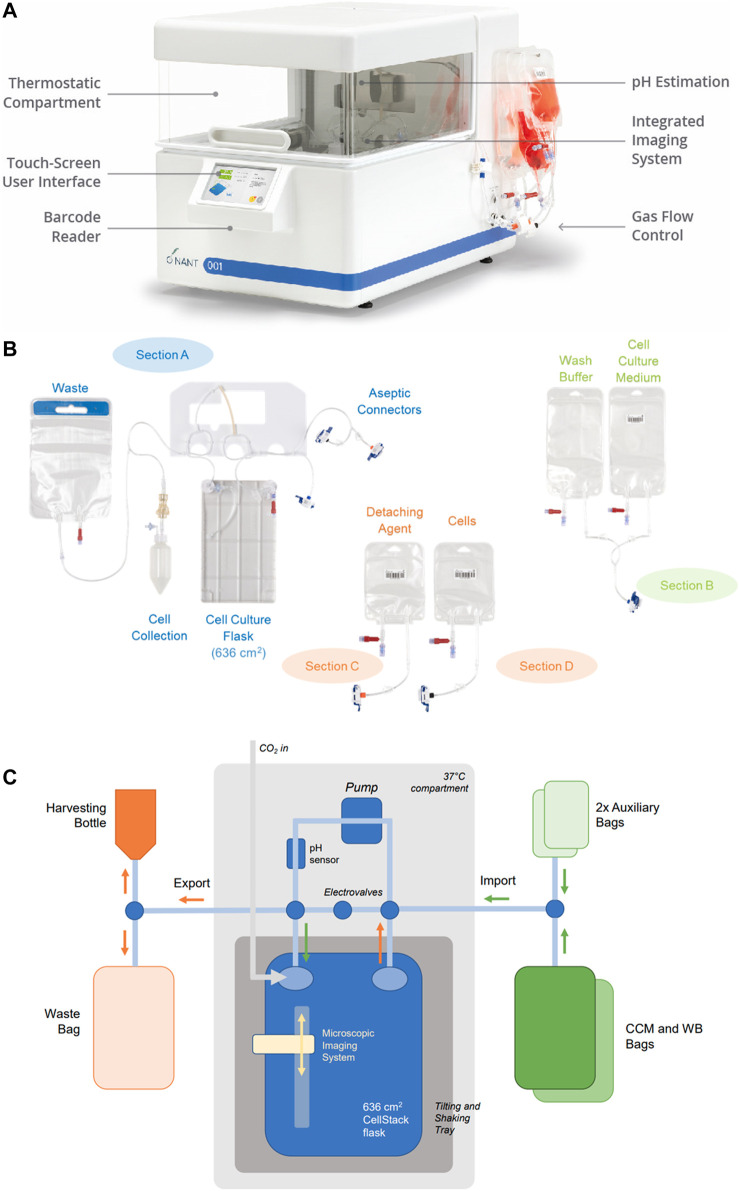
Bioreactor design. **(A)** The closed, automated NANT 001 bioreactor with thermostatically controlled compartment, touch screen interface, barcode reader and with single use fluid reservoirs attached. **(B)** Configuration of the single use components of the bioreactor, comprising of a cell culture flask, reservoirs for storage of media, wash buffer, cells prepared for seeding, detaching agent (typically an enzyme preparation), bag for waste fluids and receptable for collection of the expanded cell population. **(C)** Fluidics design of the NANT 001 bioreactor system incorporating a sterile, single-use cell-culture chamber with a tilt and shake mechanism connected in a closed system to a series of bags containing complete culture medium, wash buffer (WB), detaching agent, cell suspension for seeding, a waste bag and a harvesting bottle. Continuous monitoring of temperature and pH is achieved by the inclusion of sensors. Cell morphology and confluence are monitored using an integrated, self-operating and auto-focusing microscope.

The essential design features of the bioreactor are shown in Figure 1.

The system design incorporates several features which align with EU guidelines for GMP-compliant advanced therapy medicinal products (ATMP) manufacturing. A description of these features and the relevant sections of “Guidelines on Good Manufacturing Practice specific to Advanced Therapy Medicinal Products”, issued by the European Commission (European Commission, 2017) are given in Table 1.

**TABLE 1:** Design features of the NANT 001 bioreactor which align to relevant sections of “Guidelines on Good Manufacturing Practice specific to Advanced Therapy Medicinal Products”, issued by the European Commission” (European Commission, 2017).

Section	Content	NANT 001 design feature
9.35	Measures to prevent cross-contamination including: (iii) Use of “closed systems” for processing and material/product transfer between equipment (v) Use of single use disposable technologies	The NANT 001 bioreactor system operates without ever coming into direct contact with cells and reagents using a sterile, single-use, closed-system disposable unit (NANT cartridge)
5.13, 9.44	Connections that are to be made in aseptic conditions should be performed in a critical clean area of grade A with a background clean area of grade B, unless the connection is made by means of a validated sterile system	Validated aseptic connectors are used to connect different sections to the cartridge when required, ensuring a closed system is maintained
	When materials are added/withdrawn from the closed system without an aseptic connection the system can no longer be considered closed	
9.29	Gases taken into the aseptic workplace or that come into contact with the product should be passed through sterilising filters	The NANT Cartridges include a sterile, single-use, disposable 0.22 μm filter for air/CO_2_ to be imported to the cell culture container or into the thermostatic compartment
9.41, 17.24	Production in a closed system, in an isolator, or positive pressure isolators: a background clean area of Grade D is acceptable	Expansion of cells within the NANT 001 system occurs in a closed system enabling operation in a Grade D area
4.41	The presence of containers and/or materials liable to generate particles should be minimised in the clean areas	The NANT 001 bioreactor does not include parts that generate particles, nor parts that may spread particles
4.19 (a)	The use of more than one closed isolator (or other closed systems) in the same room at the same time is acceptable, provided that appropriate mitigation measures are taken to avoid cross-contamination or mix-ups of materials, including separated expulsion of the exhausted air from the isolators	The NANT 001 system can be operated exclusively with one NANT Cartridge per run
		Parallel cultures with two different NANT systems are totally independent
9.70	Critical quality parameters should be monitored at appropriate intervals. When technically possible, continuous monitoring of key process parameters is expected	During automated runs, key parameters pH and temperature are continuously monitored and recorded. Cell confluency is monitored and recorded at user specified intervals
		At the end of each run, a Cell Culture Report file featuring a summary of critical parameters and operator interactions can be downloaded and archived
	Any deviations should be recorded and investigated, and the measures taken should also be documented	A log file, containing all detailed parameters recorded by the system, can also be downloaded and archived
		Deviations can be manually traced

### Process

**FIGURE 2:**
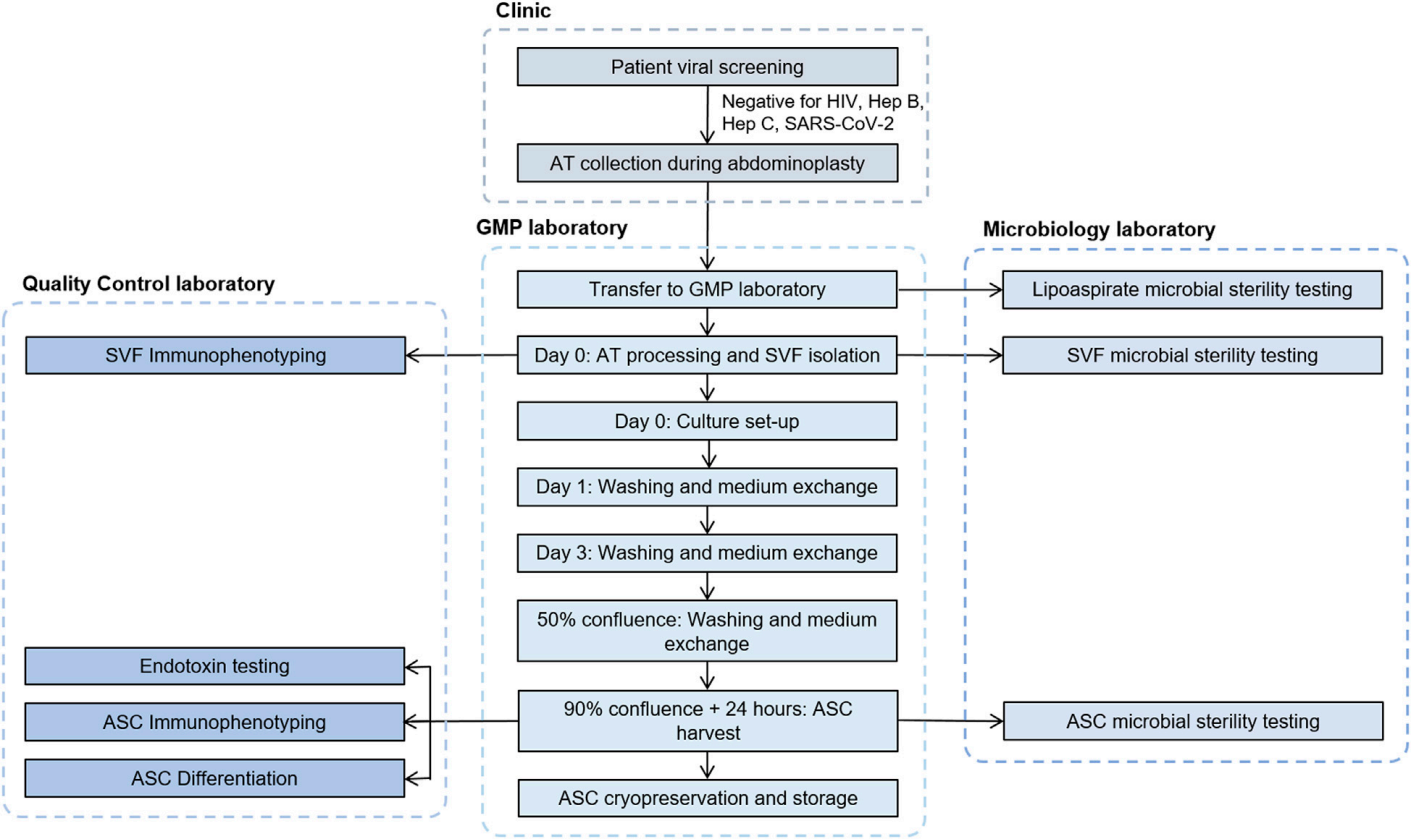
Flowchart describing the process steps and testing involved from donor qualification to cryopreservation of ASCs. ASC, adipose-derived mesenchymal stromal cells; AT, adipose tissue; GMP, good manufacturing practices; SVF, stromal vascular fraction.

The complete process from patient viral screening and donor selection to ASC cryopreservation and storage is described in detail in Figure 2.

### Materials

All reagents and materials used for isolation, expansion and cryopreservation of cells were GMP-grade certified. A complete list is provided in Supplementary Table S1.

### Donor Eligibility Criteria

Waste lipoaspirate tissues were obtained from three healthy donors (two male, one female) aged between 25 and 48, undergoing abdominoplasties at Roscommon University Hospital. The procurement of human tissues was carried out with the approval of the National University of Ireland Research Ethics Committee and the Galway University Hospital Ethics Board. In all cases the donors gave written consent. Before sample processing was initiated the donors were confirmed negative for human immunodeficiency virus (HIV) 1 and 2, hepatitis B, hepatitis C and SARS-CoV-2.

### Lipoaspirate Procurement and Isolation of SVF

Liposuction of subcutaneous abdominal adipose tissue was performed under local anaesthesia using the body-jet^®^ system (Human Med AG) to obtain a minimum of 60 ml lipoaspirate from each donor. Aspirates were transported to the manufacturing facility using an insulated transport box (Freetech) at a controlled temperature of between 2 and 8°C and stored at this temperature until processing (<24 h from the time of aspiration). Lipoaspirate was washed with Dulbecco’s phosphate-buffered saline (DPBS) and centrifuged at 340 g for 4 min following which the upper and lower phases were aseptically removed. Adipose tissue was digested by incubation with 0.4 PZ U/ml collagenase NB6 (Nordmark) in Alpha-MEM (Macopharma) at 37°C for 45 min with constant agitation. Enzymatic digestion was stopped by addition of CCM consisting of Alpha-MEM, 5% human platelet lysate (Macopharma) and 1 U/ml heparin (Wockhardt). After homogenization, the digested suspension was passed through 100 µm sterile filters, centrifuged at 600 g for 10 min, resuspended in CCM and a mononuclear cell count was obtained using a haemocytometer.

### Cell Seeding, Expansion and Harvest

Automated and manual processes were performed in parallel as follows. For the manual process, cells from the SVF were seeded in one 636 cm^2^ CellSTACK^®^ culture chamber (Corning) at a density of 4,000 cells/cm^2^ in 150 ml CCM and cultured at 37°C in an atmosphere saturated with moisture and 5% CO_2_. Washing of the cell monolayer with 150 ml DPBS and complete replacement of CCM was performed at day 1, day 3 and at 50% confluence. ASCs were harvested 24 h after the culture reached 90% confluence according to the following protocol: after aspiration of the medium and washing with DPBS, 50 ml of recombinant TrypLE solution (Gibco) was added for 5 min and incubated at 37°C. TrypLE activity was inhibited by the addition of 150 ml CCM and the cell suspension was collected.

For the automated process, the appropriate bags were filled with DPBS, CCM, TrypLE and SVF cell suspension respectively and connected to the bioreactor system via sterile connectors. The same SVF seeding density and volumes of CCM, DPBS and TrypLE as the manual process were used. Import of the CCM and SVF cells were performed by the bioreactor as well as monitoring of temperature, estimated pH and confluency. The bioreactor automatically performed all washes and media changes as described above during the expansion process. Intervention was required on the harvesting day (24 h after the system had automatically assessed the culture to have reached 90% confluency) to prompt the initiation of cell detachment and harvesting. The system performed cell detachment as described above and exported the suspended cell product to the harvesting bottle.

Samples of cell suspension from each process were taken for cell counting using a haemocytometer and assessment of viability using Trypan blue exclusion. Following counting, cell yields from each process were cryopreserved in aliquots of 2 × 10^6^/ml in 5% human albumin (Octopharma) containing 10% Cryosure-DMSO (Wak-Chemie). Samples of spent media were taken for endotoxin and microbial sterility testing.

### Cell Viability and Immunophenotype Using Flow Cytometry

Cell viability was evaluated using the Trypan Blue exclusion method and cells were manually counted under a microscope using a haemocytometer. Flow cytometry was performed to confirm cell identity in accordance with Chapter 2.7.24 of the European Pharmacopeia. ASCs at passage 0 (P0) were first washed with FACS stain buffer, passed through a 40 μm cell strainer and blocked using BD Human Fc Block™ for 10 min before incubation for 30 min at 4°C with the following fluorochrome-conjugated antibodies: anti-CD45-FITC, anti-CD90-PE, anti-CD73-APC, anti-CD14-FITC, anti-CD105-PE, anti-CD13-APC, anti-CD36-FITC, anti-CD34-PE, anti-CD31-APC. Following staining, cells were washed with stain buffer and re-suspended in 300 μl buffer for acquisition using the CytoFLEX flow cytometer. For viability assessment, cells were also incubated with 7-AAD and the data were analysed with FlowJo software.

### Differentiation Assays

The osteogenic and adipogenic propensity of ASCs cultured using automated and manual processes were assessed. For both assays, thawed P0 ASCs were seeded at 2 × 10^4^ cells/cm^2^ in 24 well plates and allowed to expand until confluent in ASC culture medium (MEM-alpha, 10% FBS). Osteogenesis was induced via incubation of ASCs in monolayer for 10–17 days in osteogenic medium (Dulbecco’s modified Eagle’s medium low-glucose [DMEM-LG], 10% FBS, 100 nM dexamethasone, 10 mM β-glycerophosphate, 100 µM ascorbic acid-2-phosphate), while control cultures were maintained in ASC culture medium. The level of osteogenesis was assessed by staining deposited calcium with alizarin red and quantification of calcium using the Stanbio Calcium (CPC) LiquiColor^®^ Test. Adipogenesis was performed by incubation of ASCs in monolayer for 3 days with adipogenic induction medium (Dulbecco’s modified Eagle’s medium high-glucose [DMEM-HG], 10% FBS, 1 µM dexamethasone, 500 µM methyl-isobutylxanthine, 10 μg/ml insulin and 200 µM indomethacin), followed by a 1 day incubation in adipogenic maintenance medium (DMEM-HG, 10% FBS and 10 μg/ml insulin). ASCs were subjected to three rounds of this cycle, with the final maintenance cycle lasting 5–7 days. Control cultures were maintained in ASC culture medium. The level of adipogenesis was assessed by staining lipid deposits with Oil Red O. The stain was then extracted via incubation with isopropanol and measured spectrophotometrically at 520 nm for quantification.

### Bacterial and Endotoxin Contamination Testing

Bacterial contamination was assessed using the Bactec FX40 system in accordance with methodology recommendations of Chapter 2.6.27 of the European Pharmacopeia (Ph. Eur.). Cell supernatants were collected and 3 ml of each were inoculated in aerobic and anaerobic culture bottles for up to 7 days. The absence of bacterial growth was considered a negative result. The presence of endotoxin was analysed from the supernatant of cell cultures following harvest using the limulus amoebocyte lysate (LAL) test. Measurements were performed in accordance with the instructions of Chapter 2.6.14 of the European Pharmacopeia. Values of ≤1 EU/ml were considered negative for endotoxin contamination.

### Media Fill

As part of the process validation, three aseptic processing ‘media fill’ runs were also performed. All solutions and reagents used in the manufacturing process were replaced by Tryptic Soy Broth (TSB) and all aseptic manipulations including filling of the bags, cell seeding, washes, media changes and harvesting were simulated with the incubation time reduced to 1 day. Sampling activities were carried out as performed for normal routine production (representative samples taken to simulate endotoxin and microbial sampling) with the additional inclusion of the waste bag which was sealed off using a heat sealer and the cell suspension bottle.

### ASC Specifications

Stringent in-process controls were set up to control the critical steps of cell expansion from receipt of lipoaspirate tissue to the final end product (P0 ASCs harvested from the bioreactor). This set of specifications was established to ensure GMP compliance and to determine if ASCs produced by the bioreactor were equivalent to those produced during traditional manual processing. Prior to tissue procurement, serological testing was performed on donors to ensure absence of hepatitis B, C and HIV. Maintenance of the lipoaspirate between +2°C and +8°C during transport was required. A minimum weight of 60 g of harvested adipose tissue was specified with processing taking place within 24 h of harvest. A minimum weight of 20 g of processed adipose tissue was required following centrifugation in order to proceed with tissue digestion. Following isolation of SVF, specifications included a cell count of ≥15 × 10^6^ cells and a surface immunophenotype as follows: CD45 and CD14 <80% and CD34 ≥5%, while expression levels of CD31, CD90 and CD73 were not specified. Release criteria of the final product were defined as a yield of ≥20 × 10^6^ cells from the bioreactor, with a cellular viability ≥80%. The specified percentage of cells positive for mesenchymal markers (CD90, CD73, CD105) was ≥90% and for hematopoietic markers (CD45 and CD14) ≤5%. There was no specification for expression levels of CD13, CD31, CD34 and CD36. Supernatants of the cell culture had to return a negative result in tests for microbial contamination and have an endotoxin level of ≤1 EU/ml. Finally, cells had to demonstrate the capacity to differentiate to both adipogenic and osteogenic lineages. These specifications are summarized in Table 2.

**TABLE 2:** Specifications and in process controls for starting material, SVF and final cell product.

Process steps	Test parameters	Analytical method	Specifications	Eur. Ph. Reference
Donor validation	Donor serological testing	PCR	Negative for Hepatitis B, C, HIV, SARS-CoV-2	n/a
Receipt of adipose tissue at the manufacturing site	Weight of adipose tissue	Weight	>60 g	n/a
	Temperature during transport	Temperature tracker	Between +2°C and +8°C	n/a
	Duration of time between harvest and processing of tissue	Monitor time	<24 h	n/a
	Weight of pure adipose tissue (following centrifugation)	Weight	>20 g	n/a
Isolation of SVF	Cell count	Haemocytometer	≥15 × 10^6^ cells	Eur. Ph. 2.7.29
	CD34^+^	Flow cytometry	≥5%	Eur. Ph. 2.7.24
	CD45^+^	Flow cytometry	<80%	Eur. Ph. 2.7.24
	CD14^+^	Flow cytometry	<80%	Eur. Ph. 2.7.24
	CD31^+^	Flow cytometry	Not specified	Eur. Ph. 2.7.24
	CD73^+^	Flow cytometry	Not specified	Eur. Ph. 2.7.24
	CD90^+^	Flow cytometry	Not specified	Eur. Ph. 2.7.24
Final product (P0 ASCs)	Cell count	Haemocytometer + trypan blue	≥20 × 10^6^ cells	Eur. Ph. 2.7.29
	Viability	Trypan blue, 7AAD	≥80%	n/a
	CD90^+^	Flow cytometry	≥90%	Eur. Ph. 2.7.24
	CD73^+^	Flow cytometry	≥90%	Eur. Ph. 2.7.24
	CD105+	Flow cytometry	≥90%	Eur. Ph. 2.7.24
	CD45^+^	Flow cytometry	≤5%	Eur. Ph. 2.7.24
	CD14^+^	Flow cytometry	≤5%	Eur. Ph. 2.7.24
	CD13^+^	Flow cytometry	Not specified	Eur. Ph. 2.7.24
	CD31^+^	Flow cytometry	Not specified	Eur. Ph. 2.7.24
	CD34^+^	Flow cytometry	Not specified	Eur. Ph. 2.7.24
	CD36^+^	Flow cytometry	Not specified	Eur. Ph. 2.7.24
	Microbial testing	Bactec	Negative	Eur. Ph. 2.6.27
	Endotoxin testing	LAL Test	≤1 EU/ml	Eur. Ph. 2.6.14

Abbreviations: Eur. Ph. (European Pharmacopoeia); PCR (polymerase chain reaction); HIV (human immunodeficiency virus); SVF (stromal vascular fraction); ASCs (adipose-derived mesenchymal stromal cells); 7AAD (7-Aminoactinomycin D); qPCR (quantitative polymerase chain reaction); LAL (limulus amoebocyte lysate).

### Labor Commitment and Cost-Effectiveness Analysis

A cost-effectiveness analysis was performed to compare the cost of an open manual manufacturing process with an automated process using the NANT 001 system for the production of an autologous MSC-based therapy. The analysis was based on the following assumptions: 300 working days per year, a production team consisting of two manufacturing technicians, one quality control technician and one Qualified Person (all FTE), one Class A/B cleanroom facility available and a production process of 8 ± 1 days resulting in the production of 130 batches per year. Upstream, downstream and all manual expansion processes were performed in a Grade A/B cleanroom with the bioreactors operating in a Grade D area. All relevant manufacturing costs including the expansion phase and all upstream and downstream processes were calculated and compared. Costs analyzed included fixed costs, both direct (facility, Capex, maintenance and inspection) and indirect (running costs, administration overheads, insurance etc.) and direct variable costs including labour (manufacturing, QC and QA/QP), consumables, reagents, cleaning and QC tests.

Based on the same assumptions, an analysis of the labor commitment required to support the automated production of a patient batch using the NANT 001 system compared to an equivalent manual production process was performed. Working hours for manufacturing technicians, quality control technician and Qualified Person were calculated per batch for both automated and manual processes.

## Results

### Research Validation

Prior to execution of the validation runs in the GMP facility, a series of expansion runs were first carried out in a process development/research laboratory to permit training of validation scientists and the generation of batch manufacturing records and standard operating procedures. These runs were conducted using the same protocol as those carried out under GMP conditions, except cells were harvested at 90% confluency. Three research validation runs were performed using pre-expanded ASCs at passage 1. Following harvest, cell viability, sterility, differentiation capacity and surface immunophenotype were assessed. In brief, cells cultured using both automated and manual processes demonstrated the characteristic fibroblast-like morphology of ASCs and cell yields were comparable from both cultures. Viability was not affected by the bioreactor process with >96% cell viability recorded from all three runs. Supernatants from all cultures tested negative for microbial contamination. Expression of surface markers in all cases was consistent with standards published by the International Federation for Adipose Therapeutics and Science (IFATS) and the International Society for Cellular Therapy (ISCT) for ASC characterization (Bourin et al., 2013). There were no significant differences in the adipogenic and osteogenic differentiation propensity of the cells regardless of the expansion process (data not shown).

### GMP Validation

#### Expansion of ASCs From Automated and Manual Processes

**FIGURE 3:**
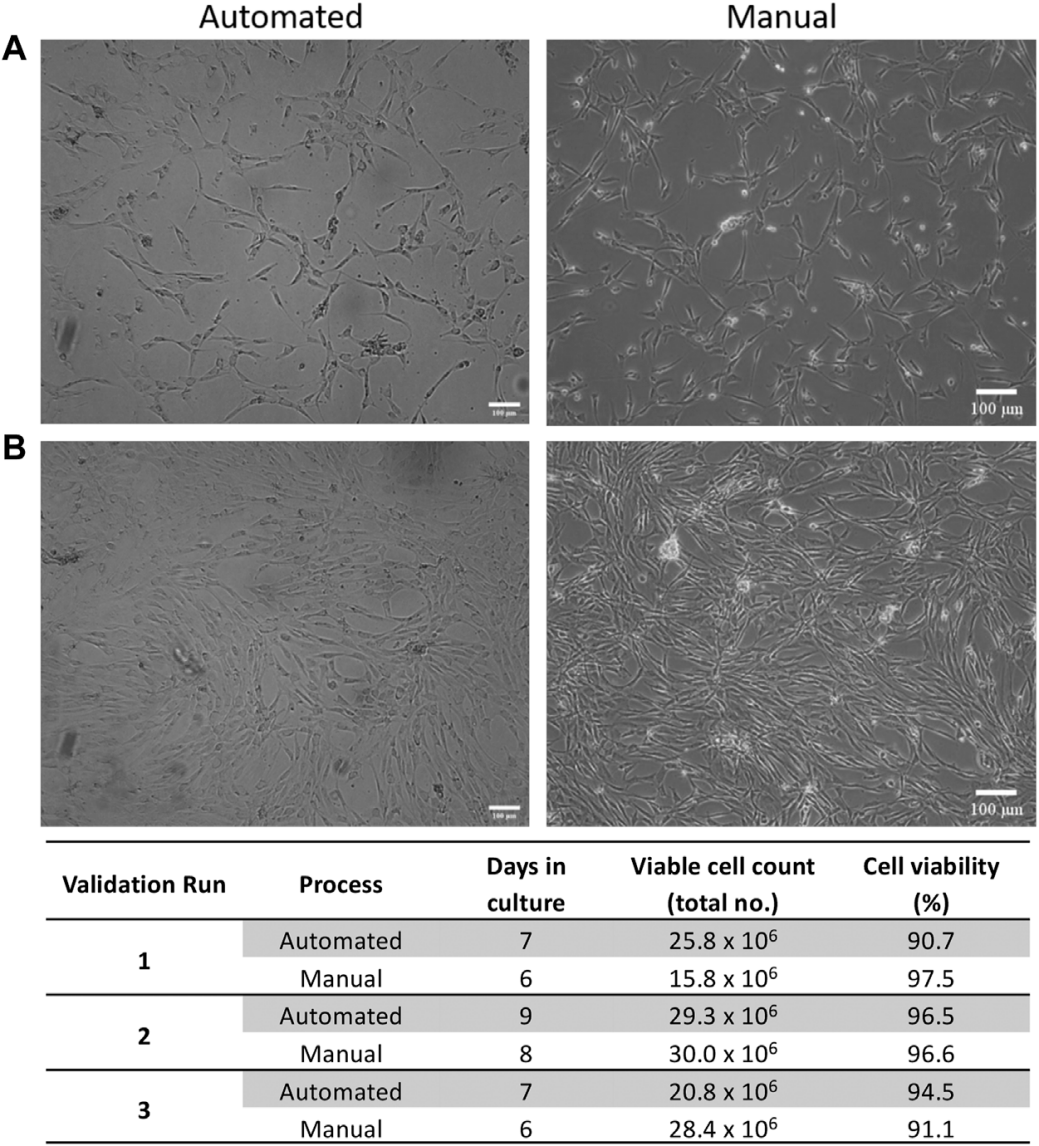
Expansion characteristics of ASCs. Representative phase contrast images of cell morphology at **(A)** 50% and **(B)** 90% confluency respectively for automated and manual processes. **(C)** Expansion characteristics of cells from automated and manual processes for each validation run. Scale bar = 100 µm.

ASCs cultured in both automated and manual processes displayed the typical fibroblastic morphology associated with ASCs (Figures 3A,B). The number of days in culture ranged from 7–9 days for the automated process and 6–8 days for the manual process. This is likely due to loading the SVF suspension into the NANT 001 bag, where some loss of SVF suspension in residual space of the tubing occurs. Cell yields from the automated processes (average 2.5 × 10^7^ ± 7.8 × 10^6^) were comparable to those from the manual process (2.5 × 10^7^ ± 4.3 × 10^6^) and cell viability was >90% for all cultures (Figure 3C).

#### Immunophenotype of ASCs From Automated and Manual Processes

**FIGURE 4:**
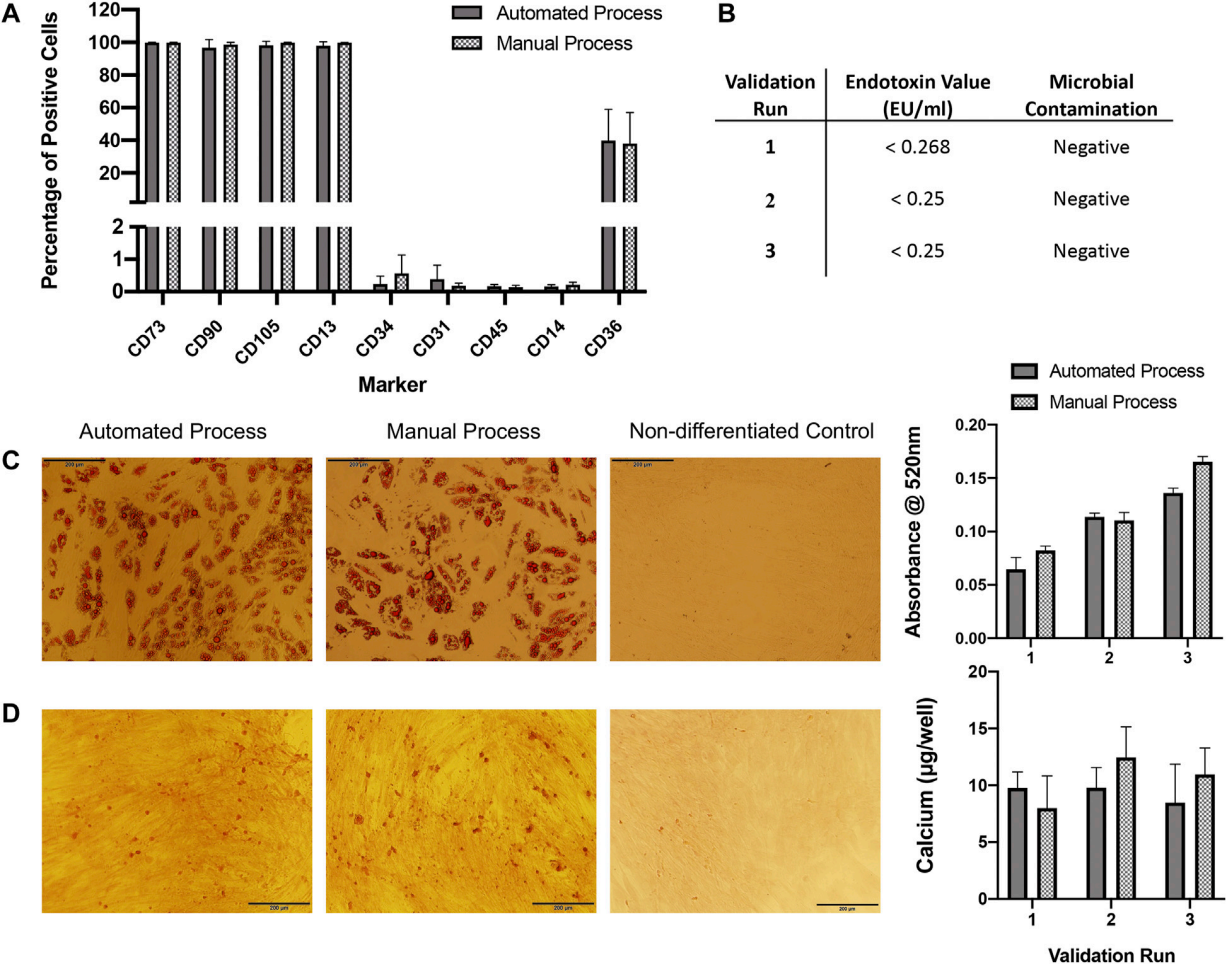
QC analysis. **(A)** Flow cytometry immunophenotyping of ASCs expanded in automated or manual processes over three validation runs (*n* = 3). Expression is shown as percentage positive cells. Results are expressed as mean ± SEM. **(B)** ASCs expanded in both automated and manual cultures tested negative for microbial contamination. Endotoxin concentration was assessed in automated cultures only and was <1 EU/ml for all. **(C)** ASCs expanded using the automated and manual processes were capable of undergoing adipogenic differentiation for all three validation runs, representative images of oil red O staining in automated and manual process test samples and a control (non-differentiated) sample are shown. Semi-quantification of oil red O staining by measurement of absorbance at 520 nm was performed, results are expressed as mean ± SEM. Magnification = ×10, scale bar = 200 µm. **(D)** ASCs expanded using the automated and manual processes were capable of undergoing osteogenic differentiation for all three validation runs, a representative image of alizarin red staining in automated and manual process test samples and a control sample are shown. Calcium quantification was performed for each validation run, results are expressed as mean ± SEM. Magnification = ×10, scale bar = 200 µm. Calcium deposition was not detected in control wells (data not shown).

ASC immunophenotypes were consistent with the IFATS and ISCT standard. Surface marker expression profiles were almost identical between expansion processes (Figure 4A). More than 90% of ASCs from both processes were positive for CD73, CD90, CD105 and CD13. Expression of CD36 varied between donors but was consistent between processes, approximately 20–60% of cells expressed CD36. Expression of the standard negative markers CD31, CD45 and CD14 was less than 1% in all cases.

#### Sterility Tests

Microbial tests for aerobic and anaerobic bacteria from automated and manual cultures were all negative. Endotoxin concentration was assessed for automated cultures only and was <1 EU/ml for all cell preparations (Figure 4B).

#### Differentiation Potential of ASCs

ASCs expanded using both automated and manual processes were tested for their osteogenic and adipogenic differentiation propensity. ASCs from all three validation runs were capable of osteogenic and adipogenic differentiation (Figures 4C,D).

### Labor Commitment and Cost-Effectiveness Analysis

**FIGURE 5:**
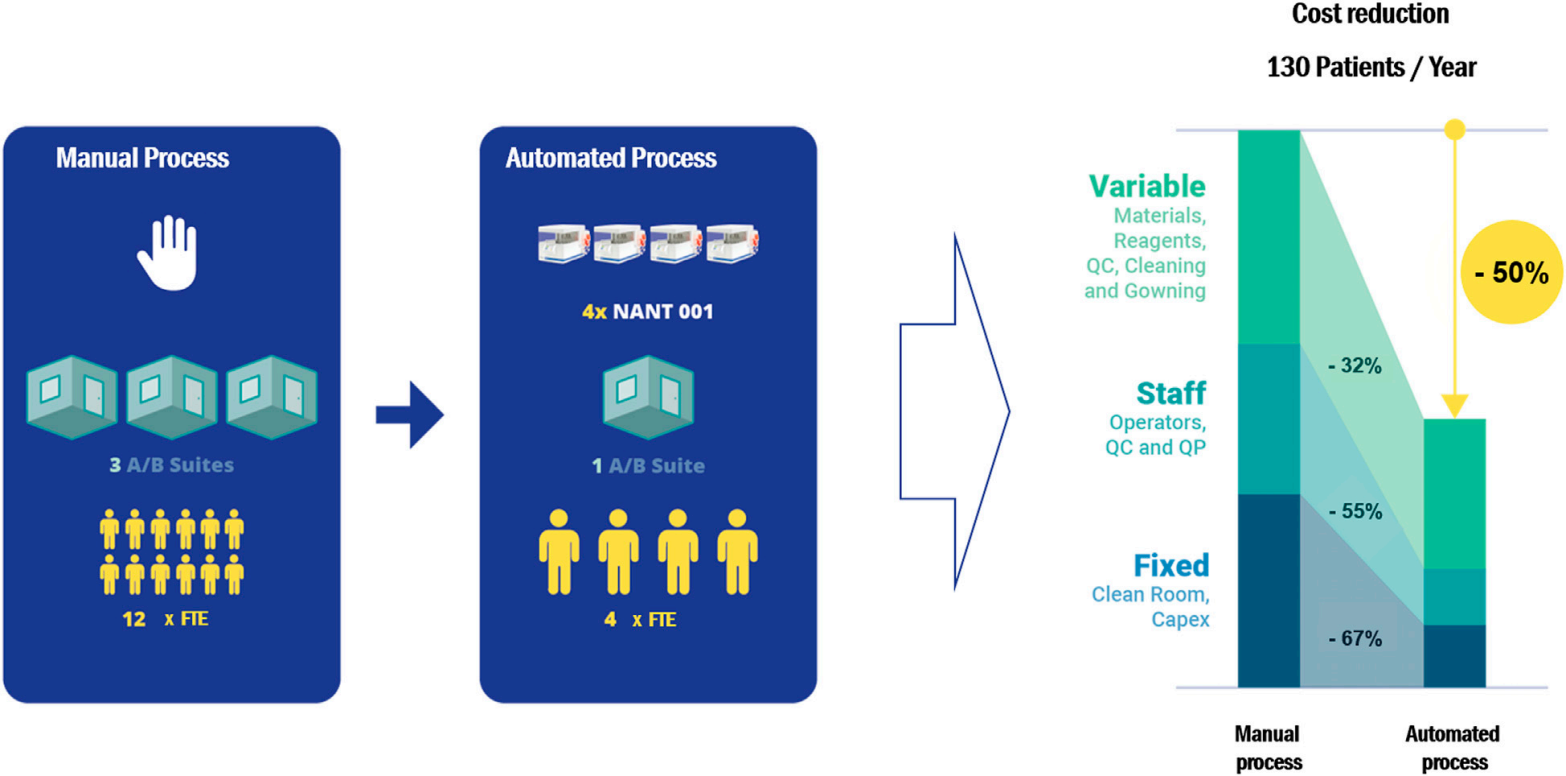
A cost-effectiveness analysis comparing the cost of a manual expansion process with an automated process using the NANT 001 system to produce 130 batches of an autologous ASC therapy per year. The following assumptions were applied: personnel consists of a production team of two manufacturing technicians, one QC technician and one Qualified Person (all FTE), one Class A/B cleanroom facility available and a production process of 7 ± 1 days. Upstream, downstream and all manual expansion processes would be performed in a Grade A/B cleanroom with the bioreactors operating in a Grade D area. All relevant manufacturing costs including direct and indirect fixed costs and direct variable costs for the expansion phase and all upstream and downstream processes were calculated and compared.

Cost-effectiveness analysis of the automated process demonstrated an overall cost reduction of 50% compared to equivalent manual processing, based on the production of 130 batches per year (Figure 5). Reductions in variable, fixed and personnel costs were 32, 67 and 55% respectively. A detailed breakdown of the cost analysis is given in Supplementary Table S2.

**FIGURE 6:**
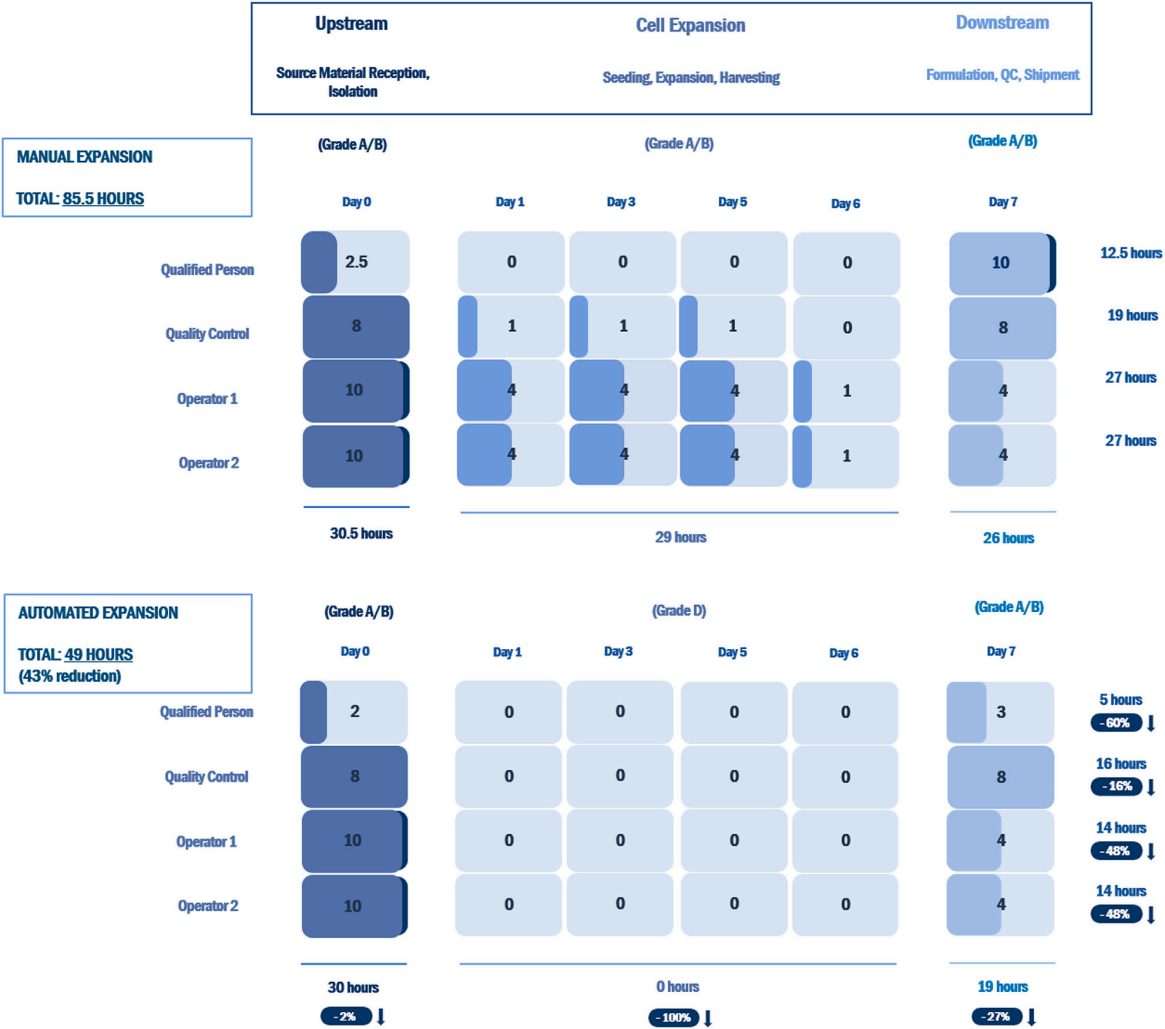
Labor commitment analysis comparing an open, manual expansion process with an automated expansion process using the NANT 001 system for an autologous therapy with a production process of 7 ± 1 days. Results are presented as hours of labor required per batch by a production team consisting of two manufacturing technicians, one QC technician and one Qualified Person for all upstream, downstream and expansion processes. The percentage reductions in hours of labor for the automated expansion process vs. the manual process are indicated.

Analysis of the labor commitment indicates considerable savings with automated processing in terms of hours of labor per batch, with an overall reduction of 43% over an equivalent manual process (Figure 6). Automated feeding reduces the labor burden on production operators by 48%, while the associated reduction in QC testing reduces QC labor by 16%. The reporting performed by the bioreactor decreases the labor required by the Qualified Person by 60%.

## Discussion

The production of expanded MSCs from post-natal sources for therapeutic use is a complex process, requiring stringent control of the aseptic environment. In traditional manual processes, that are essentially open, cells and other materials are exposed to the environment. This requires a dedicated and fully GMP-compliant facility with physical infrastructure elements including air exchange, air filtration, barriers and controlled access. In addition, an aseptic processing facility represents a sub-optimal work environment with a risk to operators of fatigue and exposure to repetitive manipulation. It also involves a measurable risk of operator-associated or environmental contamination. As the manufacture of cell therapy products increases dramatically, these considerations become critically important. In this context, the advantage of a fully closed expansion system is self-evident in terms of product safety and operational convenience.

Several automated systems have been developed and marketed in recent years for MSC expansion, incorporating a wide array of bioreactor configurations. These include the Quantum Cell Expansion System, a closed automated hollow-fibre bioreactor developed by Terumo which has been widely used for the large-scale expansion of MSCs (Rojewski et al., 2013; Russell et al., 2018). This system provides advantages in terms of process scalability and is adaptable to a range of different cell types, viral vectors and exosomes. For adherent cells however, an attachment factor is required, the selection of which can greatly affect the cell yield (Frank et al., 2019), while harvesting of expanded adherent cells from the hollow fibre material may be quite challenging. Furthermore, the scale of this system precludes its use in autologous manufacturing.

The CliniMACS Prodigy^Ⓡ^ developed by Miltenyi Biotec consists of a closed system of interlinked bioreactors and bioprocessors designed for large-scale end-to-end production (Zhu et al., 2018). They offer two platform configurations, the T-Cell Transduction Process for the automated generation of engineered T cells, primarily chimeric antigen receptor (CAR)-T cells (Miltenyi Biotec, 2020a). The Adherent Cell Culture System, is an all-in-one solution which can perform isolation of MSCs from bone marrow by density gradient centrifugation, cell expansion, subculture and harvesting (Miltenyi Biotec, 2020b). However, for small-scale ASC manufacturing, this may represent an overly complex and expensive manufacturing solution given the high cost of associated consumables (Dai et al., 2019).

The Cocoon system presented by Lonza provides a more compact solution for end-to-end production in a manner which, similar to the NANT 001, accommodates scale out with application at the point of care (Byrne, 2020). The platform is customizable, with multiple functional modules including isolation, cell selection, activation, transduction and expansion (Lonza, 2021b). Lonza have recently reported the treatment of four patients with B-cell malignancies using CD19 autologous CAR-T cells expanded using the Cocoon platform (Lonza, 2021a). This is potentially a very useful solution for the expansion of MSCs but its wide adoption at this time is unclear.

Another bioreactor option that is widely used is the Xuri Cell Expansion System W25 from Cytiva, a closed, automated system based on WAVE™ rocking technology (Cytiva, 2021). It has been primarily used for the large-scale production of suspension cells such as T cells, CAR-T cells and natural killer cells (Smith, 2020) but has also been tested for the expansion of adherent cell types including MSCs on microcarriers or in aggregates (Tsai et al., 2017; Davis et al., 2018).

The NANT 001 solution is ideally scaled for autologous use making it suitable for use in a distributed manufacturing model. It has been designed to perfectly reproduce manual cell culture processes (Paulitti et al., 2020), is compatible with all standard culture reagents and does not require the use of attachment factors for MSCs, enabling an easy translation of manual processes to an automated expansion process incorporating the system. The imaging system is a unique feature which provides visual feedback to the operator and constant monitoring of the culture. At the end of the process, complete traceability of all operations and events is guaranteed through the generation of a detailed cell culture report.

The results of this study provide several interesting findings: 1) the NANT 001 bioreactor is capable of producing ASCs with identical phenotype and yield compared to standard manual processing; 2) the bioreactor is fully compatible with a GMP environment; 3) it eliminates a large number of manual processing steps, greatly reducing the labor burden per patient batch and the risk of contamination; 4) it provides valuable real time analytical information on cell health, growth and morphology; 5) it contributes to a significant reduction of cost of goods.

A number of critical quality parameters have been suggested for cell therapy products, including product identity, purity and safety (Robb et al., 2019). The cells isolated and produced by the NANT 001 bioreactor in this study were in accordance with these critical quality attributes in that cell viability was >90%, surface marker expression profiles were consistent with IFATS standards (Bourin et al., 2013), cells were free from microbial and endotoxin contamination and were capable of differentiating towards the adipogenic and osteogenic lineages.

Finally, being a completely automated and closed system, the NANT 001 can be effortlessly operated in a Grade D environment, and utilised in a multiplex configuration, with several instruments operating in parallel increasing the efficiency of the facility and process workflow, and ultimately resulting in a reduction of cell therapy manufacturing costs. All these results confirm the ability of the NANT 001 System in conducting automated ASC expansions, meeting all the GMP requirements in terms of safety, traceability and reproducibility in a cost-effective manner.

## Data Availability

The original contributions presented in the study are included in the article/Supplementary Material, further inquiries can be directed to the corresponding author.
