# Supplementary material for: GMP-Compliant Production of Autologous Adipose-Derived Stromal Cells in the NANT 001 Closed Automated Bioreactor
Source: Front Bioeng Biotechnol. 2022 Mar 9;10:834267. doi: 10.3389/fbioe.2022.834267 (PMC8959900; doi:10.3389/fbioe.2022.834267)
Supplement: Supplementary file 1 [file DataSheet1.docx]

**Supplementary Table 1: Bill of materials used for the isolation, expansion and cryopreservation of cells.**

| **Material** | **Supplier** | **Code** | **Specification** |
| --- | --- | --- | --- |
| MEM α, GlutaMAX™ Supplement, no nucleosides | Gibco | 32561029 | Certificate of  analysis |
| Platelet Lysate hPL100 | Macopharma | BC0190020 | Certificate of analysis |
| Heparin PL | Wockhardt | 29831/0110 | For i.v.  human use  (or  subcutaneous) |
| Acetic Acid ≥99.99% | Sigma-Aldrich | 8187551000 | Certificate of analysis |
| Phosphate Buffered Saline Solution (PBS) | Macopharma | 0120020 | Certificate of analysis |
| TrypLE™ Select CTS™ | Gibco | A1285901 | Certificate of analysis |
| Collagenase | Nordmark | 001745803 | Manufactured in compliance with Good Manufacturing Practice (GMP); certificate of analysis |
| Albunorm 5% | Octapharma | PA521/16/1 | For i.v. human  use |
| BD Bactec Peds Plus Aerobic/F Bottle | Becton Dickinson | 442020 | CE marked; certificate of quality |
| BD Bactec Plus Aerobic/F Bottle | Becton Dickinson | 442023 | CE marked; certificate of quality |
| BD Bactec Lytic 10 Anaerobic/F Medium Bottle | Becton Dickinson | 442021 | CE marked; certificate of quality |
| 1 Layer CellSTACK® | Corning | CLS3303 | Certificate of analysis |
| 100 µm Cell Strainer | Falcon Brand | 352360 | Certificate of quality |
| Origen Needle Free Spike – DMSO Resistant | OriGen Biomedical | CBS | CE marked |
| BD 1 ml Syringe | Becton Dickinson | 309628 | CE marked;  certificate of  compliance |
| BD 10 ml syringe | Becton Dickinson | 300912 | CE marked;  certificate of  compliance |
| BD 50 ml Syringe | Becton Dickinson | 300865 | CE marked;  certificate of  compliance |
| Sterile Needles 23G | Becton Dickinson | 300800 | Certificate of compliance |
| Nalgene® cryogenic 5 ml vials | Sigma-Aldrich | V5257 | Certificate of analysis |
| Centrifuge Tube 50 ml | Corning | CLS430290 | Certificate of analysis |
| Nunc Serological 1 ml Pipette | Nunc | 170353 | Certificate of conformity |
| Nunc Serological 5 ml Pipette | Nunc | 170355 | Certificate of conformity |
| Nunc Serological 10 ml Pipette | Nunc | 170356 | Certificate of conformity |
| Nunc Serological 25 ml Pipette | Nunc | 170357 | Certificate of conformity |
| Nunc Serological 50 ml Pipette | Nunc | 170358 | Certificate of conformity |
| Nunc 1 ml Cryovials | Nunc | V7384 | CE marked;  certificate of  analysis |
| 500 ml PET Bottles | Corning | 431733 | Certificate of quality |
| FOS | Becton Dickinson | 442153 | *In vitro* diagnostic use; certificate of quality |
| Wak-Chemie CryoSure DMSO | WAK-Chemie Medical GmbH | WAK-DMSO-50 | CE 0482 |

**Supplementary Table 2: Cost Analysis**

|  | **MANUAL PROCESS** | **NANT 001 SYSTEM** | **SAVINGS** |
| --- | --- | --- | --- |
| Number of cleanroom units | 3 | 1 |  |
| Total cleanroom space - GRADE B (depreciation) | 360,000.00 € | 85,714.29 € |  |
| Maintenance and inspection costs | 144,000.00 € | 48,000.00 € |  |
| CAPEX Equipment (depreciation) | 68,142.86 € | 20,000.00 € |  |
| NANT 001 Bioreactors (depreciation) | - € | 34,285.71 € |  |
| **Direct Fixed Costs (cleanroom, lab equipment, bioreactors)** | 572,142.86 € | 188,000.00 € |  |
| Indirect Fixed Costs (bills, insurance, admin overheads, etc.) | 108,000.00 € | 36,000.00 € |  |
| **Total facility costs** | **680,142.86 €** | **224,000.00 €** | **-67%** |
|  |  |  |  |
| **Staff (Direct Labour)** |  |  |  |
| Operators | 266,220.00 € | 110,040.00 € |  |
| QC | 92,340.00 € | 62,880.00 € |  |
| QA and QP | 76,950.00 € | 24,776.00 € |  |
| **Total staff costs (operators, QC, QA, QP)** | **435,510.00 €** | **197,696.00 €** | **-55%** |
| Patients | 130 | 130 |  |
| Doses | 130 | 130 |  |
| Production batches | 130 | 130 |  |
| **Manufacturing (upstream, expansion, downstream, QC)** | **746,796.45 €** | **510,075.52 €** | **-32%** |
| Total direct variable cost | 1,182,306.45 € | 707,771.52 € |  |
|  |  |  |  |
| **Fixed costs/dose** | 5,191.93 € | 1,709.92 € |  |
|  |  |  |  |
| **Variable cost/dose** | 9,025.24 € | 5,402.84 € |  |
|  |  |  |  |
| **Manufacturing (upstream, expansion, downstream, QC)/dose** | 5,700.74 € | 3,893.71 € |  |
|  |  |  |  |
| **Total cost/year** | 1,862,449.31 € | 931,771.52 € |  |
|  |  |  |  |
| **Total cost/dose** | **14,217.17 €** | **7,112.76 €** | **-50%** |
